# Identification of Diagnostic CpG Signatures in Patients with Gestational Diabetes Mellitus *via* Epigenome-Wide Association Study Integrated with Machine Learning

**DOI:** 10.1155/2021/1984690

**Published:** 2021-05-19

**Authors:** Yan Liu, Hui Geng, Bide Duan, Xiuzhi Yang, Airong Ma, Xiaoyan Ding

**Affiliations:** ^1^Department of Obstetrics, Tianjin First Central Hospital, Nankai University, Tianjin 300192, China; ^2^Department of Obstetrics, Zibo Central Hospital, Zibo City, Shandong Province, 255000, China

## Abstract

**Background:**

Gestational diabetes mellitus (GDM) is the most prevalent metabolic disease during pregnancy, but the diagnosis is controversial and lagging partly due to the lack of useful biomarkers. CpG methylation is involved in the development of GDM. However, the specific CpG methylation sites serving as diagnostic biomarkers of GDM remain unclear. Here, we aimed to explore CpG signatures and establish the predicting model for the GDM diagnosis.

**Methods:**

DNA methylation data of GSE88929 and GSE102177 were obtained from the GEO database, followed by the epigenome-wide association study (EWAS). GO and KEGG pathway analyses were performed by using the clusterProfiler package of R. The PPI network was constructed in the STRING database and Cytoscape software. The SVM model was established, in which the *β*-values of selected CpG sites were the predictor variable and the occurrence of GDM was the outcome variable.

**Results:**

We identified 62 significant CpG methylation sites in the GDM samples compared with the control samples. GO and KEGG analyses based on the 62 CpG sites demonstrated that several essential cellular processes and signaling pathways were enriched in the system. A total of 12 hub genes related to the identified CpG sites were found in the PPI network. The SVM model based on the selected CpGs within the promoter region, including cg00922748, cg05216211, cg05376185, cg06617468, cg17097119, and cg22385669, was established, and the AUC values of the training set and testing set in the model were 0.8138 and 0.7576. The AUC value of the independent validation set of GSE102177 was 0.6667.

**Conclusion:**

We identified potential diagnostic CpG signatures by EWAS integrated with the SVM model. The SVM model based on the identified 6 CpG sites reliably predicted the GDM occurrence, contributing to the diagnosis of GDM. Our finding provides new insights into the cross-application of EWAS and machine learning in GDM investigation.

## 1. Introduction

Diabetes mellitus is a heterogeneous disorder of metabolic diseases described by hyperglycemia occurring from defects in insulin resistance or insulin secretion [1]. Gestational diabetes mellitus (GDM) is one of the most prevalent pregnancy complications, with incidence estimates varying from 2% to 25% depending on the diagnostic criteria used and the population measured [2, 3]. In addition to the adverse pregnancy and delivery outcomes correlated with GDM, which can include shoulder dystocia, macrosomia, and preeclampsia [4], women diagnosed with GDM are four times more likely to have children who acquire metabolic disease later in life and twice as likely to own children who become obese or overweight [5]. Hence, there is a definite clinical need to better detect and predict GDM early in pregnancy, preventing further harm to the mother and child [6]. Although the progression of GDM has been investigated for decades, the practical and well-designed diagnostic models for the clinical prediction of GDM are extremely limited [6]. Therefore, it is essential to find effective biomarkers for improving the diagnosis and alleviating the adverse pregnancy outcomes of GDM [7, 8]. However, the advancement of this research field is still poor.

Epigenetic modification displays fundamental function in multiple processes, from molecular mechanisms to clinical application and even in biomedical transformation, and epigenetic marks are required for the pathogenesis, prevention, and diagnosis of many diseases [9, 10]. Epigenetic biomarkers provide potential signatures for the diagnosis, prognosis, and treatment of GDM as well [11, 12]. DNA methylation, mostly the CpG methylation, is the most widely studied epigenetic modification in cellular processes, providing informative alterations for the regulation of gene expression in the physiology and pathology status and serving as the potential biomarkers [13–15]. Previous studies also provide some evidence that the CpG methylation participates in the development of GDM. It has been reported that GDM is associated with genome-wide CpG methylation variation in the placenta and cord blood of exposed offspring [16, 17]. The CpG methylation profiles are also performed in adipose tissues of pregnancies with GDM [18, 19]. Besides, a genome-wide DNA methylation profile in infants born from GDM pregnancy indicates that GDM has epigenetic effects on cardiovascular disease, hypertension, diabetes, and obesity of later fetal life [20]. Thus, the exploration of CpG methylation sites may benefit the early diagnosis of GDM. However, the particular CpG sites as the reliable diagnosis biomarkers of GDM remain unclear. Epigenome-wide association study (EWAS) is a practical tool to identify the epigenetic marks associated with disease [21], and some studies have presented the role of DNA methylation in GDM development by using EWAS [22, 23]. Nevertheless, the application of EWAS in investigating the diagnostic signatures of GDM is still limited.

Machine learning is a category of information science that trains the computer to execute tasks by recognizing patterns in massive datasets and using them to determine rules or algorithms that optimize task achievement [24]. It has been reported that machine learning is beneficial to the discovery of predictive biomarkers and diagnosis of GDM [7, 25–27]. Support vector machine (SVM) is a practical machine learning model and has been confirmed to be useful classifiers in all kinds of fields, including face recognition, handwritten digit recognition, text classification, and bioinformatics [28]. Previous studies showed that SVM is widely used in the prognosis and diagnosis of multiple diseases such as cancer and diabetes mellitus [29–31]. Moreover, it has been revealed that SVM is applied in the investigation of diagnostic markers in GDM patients based on transcriptome-wide gene expression [32]. However, the practices of SVM models in the identification of CpG methylation biomarkers in the diagnosis of GDM remain unreported.

In this study, we were interested in integrating EWAS and machine learning to identify the CpG sites related to GDM. We discovered several specific CpG methylation sites that may serve as effective biomarkers of GDM and established the reliable SVM model based on the identified CpG sites for predicting the occurrence of GDM, benefiting the diagnosis of GDM.

## 2. Materials and Methods

### 2.1. Data Collection

Two DNA methylation datasets GSE88929 and GSE102177 with clinical information were downloaded from the GEO database (http://www.ncbi.nlm.nih.gov/geo/), both of which were measured by the Illumina HumanMethylation450 BeadChip assays. The GSE88929 dataset contained 68 umbilical cord blood samples from the newborns of mothers with GDM and 64 controls without GDM [12]. The GSE102177 dataset consisted of the peripheral blood samples from 18 fullsibling pairs that were exposed to different conditions of intrauterine hyperglycemia (GDM pregnancy or non-GDM pregnancy). Therefore, there were 18 samples with exposure to maternal GDM and 18 controls without exposure to GDM in the GSE102177 dataset [23].

### 2.2. Methylation Data Processing

The methylation *β*-values of the normalized CpG sites in GSE88929 and GSE102177 were downloaded. Two sets of DNA methylation data, respectively, containing 132 and 36 samples were used in this study, and the CpG sites with missing values or in the sex chromosomes were removed [33]. Then, an R package *minfi* was used to assess the quality of the CpG sites, and the CpG sites with a detection *P* value > 0.01 were removed from the analysis [34].

### 2.3. Epigenome-Wide Association Study (EWAS)

The epigenome-wide association study was carried out to investigate the relationship of methylation levels of CpGs and the GDM by using a CpGassoc R package [35, 36]. Briefly, The CpGassoc function established fixed or mixed-effects models between the GDM and methylation of individual CpG sites across the genome using a matrix or data frame of *β*-values as input. The significance was assessed by chi-squared tests, and the *P* values were calculated. The Manhattan plots were constructed to show the epigenome-wide association analysis of GDM and CpG methylation and to identify the significant CpG sites in GSE88929 with the threshold of *P* < 0.001.

### 2.4. GO and KEGG Analyses

Gene Ontology (GO) and Kyoto Encyclopedia of Genes and Genomes (KEGG) pathway analyses were performed by using the clusterProfiler package of R [37]. The GO included molecular function (MF), biological process (BP), and cellular component [5]. *P* < 0.05 was regarded as statistically significant.

### 2.5. PPI Analysis

The protein-protein interaction (PPI) analysis was constructed in the Search Tool for the Retrieval of Interacting Genes/Proteins (STRING) database (https://string-db.org/cgi/input.pl) with the threshold of confidence score ≥ 0.4 [38]. The visualization of the PPI network was presented by Cytoscape software [39].

### 2.6. SVM Models

Machine learning was performed based on the SVM model by using the e107 R package. We randomly separated the samples from GSE88929 into the training set and testing set, containing 66 samples, respectively. The samples from GSE102177 were used as the independent validation set. We established the SVM model using the *β*-values of the selected CpG sites from EWAS to assess whether the samples were GDM, in which the *β*-values of CpG sites served as the predictor variable and the occurrence of GDM served as the outcome variable. The receiver operating characteristic curve was generated to evaluate the sensitivity and specificity of the models, and the area under the curve (AUC) was calculated to assess the accuracy of the models [40–43].

## 3. Results

### 3.1. Identification of Specific GDM-Associated CpG Methylation Sites

**Figure 1:**
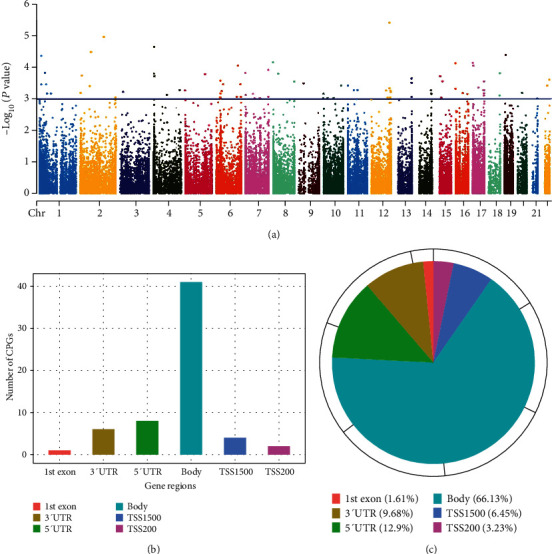
Identification of specific GDM-associated CpG methylation sites. (a) The Manhattan plot showed the association of gestational diabetes mellitus with CpG methylation in the epigenome-wide association studies of GSE88929. The *x*-axis was the location of each site across the genome. The *y*-axis was the –log_10_ of *P* value. The blue line indicated the significance threshold of *P* < 0.001. (b) The numbers among 62 identified CpG sites in the genomic region were presented in the bar diagram. (c) The distribution percentage of 62 identified CpG sites in the genomic region was demonstrated in the pie chart.

It has been identified that epigenetic regulation, such as DNA methylation or CpG methylation, is closely relative to gestational diabetes mellitus [21, 44]. However, the specific CpG sites with potential to become the biomarkers of the patient with GDM remain elusive. Epigenome-wide association study (EWAS) serves as a practical tool to study the function of DNA methylation in physiological and pathological processes such as GDM [5, 45]. In this study, DNA methylation microarray data were obtained from the GEO database, in which GSE88929 contained 68 GDM fetal cord blood samples and 64 matched normal samples. Therefore, we tried to identify the CpG sites related to GDM by EWAS in these samples. Remarkably, EWAS analysis identified that the methylation of 89 high-quality CpG sites (*P* < 0.001) was significantly changed in the GDM samples compared with the control samples after DNA methylation processing (Figure 1(a)), in which 62 CpG sites contained the gene annotation (Table S1), implying that these CpG sites may be potentially related to the development of gestational diabetes mellitus. Genomic distribution analysis further revealed that 41 among 62 CpG sites were located in the body region, and 6 among 62 CpG sites were distributed in the promoter region (TSS1500 and TSS200), accounting for 66.13% and 9.69%, respectively (Figures 1(b) and 1(c)). And other identified CpG sites were located in the exon, 3′ untranslated (3′ UTR), and 5′ UTR (Figures 1(b) and 1(c)).

### 3.2. GO and KEGG Analyses

**Figure 2:**
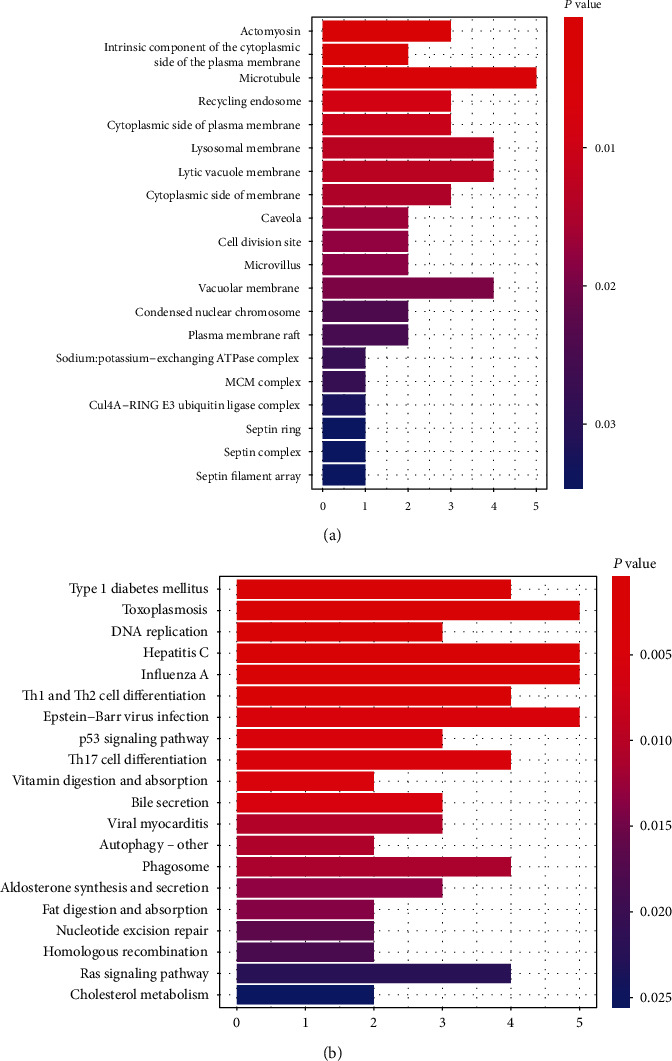
GO and KEGG analyses. (a, b) The Gene Ontology (GO) and Kyoto Encyclopedia of Genes and Genomes (KEGG) analyses were performed by using the clusterProfiler package of R. The top 20 significant cellular processes and signaling pathways were demonstrated by GO (a) and KEGG (b) enrichment analyses. The *y*-axis was the name of cellular processes or signaling pathways, and the *x*-axis was the number of genes.

For primary comprehensions of the CpG methylation-related genes, Gene Ontology (GO) and Kyoto Encyclopedia of Genes and Genomes (KEGG) pathway analyses were performed by using the clusterProfiler package of R. A total of 77 significant GO terms and 31 KEGG pathways were revealed based on the 62 identified CpG sites (Table S2), in which the top 20 remarkable GO terms and KEGG pathways were demonstrated (Figures 2(a) and 2(b)). GO analysis displayed several essential biological processes, such as sodium:potassium−exchanging ATPase complex and Cul4A−RING E3 ubiquitin ligase complex (Figure 2(a)). Besides, KEGG analysis revealed that multiple crucial signaling pathways, containing Type I diabetes mellitus, Ras signaling pathway, p53 signaling pathway, and autophagy, were enriched (Figure 2(b)).

### 3.3. PPI Network Construction

To further explore the essential CpG methylation-related genes correlated with GDM, we constructed a protein-protein interaction (PPI) network based on the 60 identified CpG sites-related genes in the STRING online database (https://string-db.org/cgi/input.pl) and Cytoscape software. Significantly, we observed 12 critical genes, including C7orf50, RASA3, PPFIA1, CASKIN1, CUL4A, POLE, MCM5, TUBGCP3, JAKMIP1, TYK2, C17orf70, and EME2, in the PPI network based on the threshold of confidence score ≥ 0.4 (Fig S1), indicating that these genes may be closely associated with the progression of GDM.

### 3.4. SVM Model Establishment

**Figure 3:**
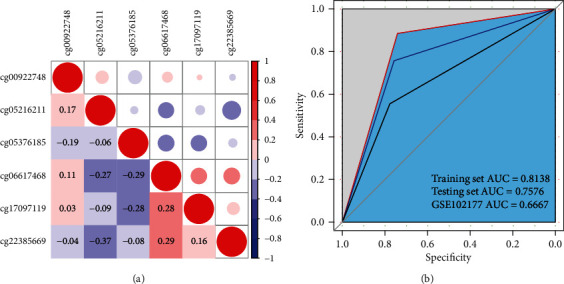
SVM model establishment. (a) Correlation matrix presented the collinearity of *β*-values of the 6 CpG sites, containing cg00922748, cg05216211, cg05376185, cg06617468, cg17097119, and cg22385669, by using collinearity analysis in GSE88929. The color and area of the circle represented the collinearity, Pearson's correlation coefficient. (b) The receiver operating characteristic (ROC) curve showed the performance of the SVM model based on the *β*-values of the 6 CpG sites, including cg00922748, cg05216211, cg05376185, cg06617468, cg17097119, and cg22385669. The *x*-axis and the *y*-axis were specificity and sensitivity, respectively. Accuracy was evaluated by the area under the curve (AUC). The red line was the training set (GSE88929, AUC = 0.8138), the blue line was the testing set (GSE88929, AUC = 0.7576), and the black line was the independent validation set (GSE102177, AUC = 0.6667).

It has been recognized that the DNA methylation within the promoter region plays a crucial role in the modulation of gene expression [46, 47]. Our EWAS analysis identified that 6 CpG sites, including cg00922748, cg05216211, cg05376185, cg06617468, cg17097119, and cg22385669, were located in the promoter region. Hence, we tried to establish an SVM diagnostic model by using the *β*-values of the identified 6 CpG sites. Firstly, our data showed that there was no strong collinearity of *β*-values of the 6 CpG sites (Figure 3(a)), providing the rationality for SVM-based machine learning by using the *β*-values of these 6 CpG sites. We randomly separated the samples from GSE88929 into the training set and testing set, containing 66 samples, respectively (Table S3). Accordingly, we constructed an SVM model, in which the predictor variable was the *β*-values of these 6 CpG sites in GSE88929, and the outcome variable was the occurrences of GDM. Surprisingly, the area under the ROC curve (AUC) analysis showed the AUC values of the training set and testing set in the model were 0.8138 and 0.7576, respectively (Figure 3(b)). Moreover, we obtained DNA methylation microarray data of 18 pairs of peripheral blood of siblings from GDM or no GDM pregnancies in GSE102177 of the GEO database, which was used as an independent validation set for the SVM model. Importantly, the AUC value of this independent validation set was 0.6667 (Figure 3(b)), suggesting that this SVM model is reliable and accurate for predicting the occurrences of GDM and may benefit the diagnosis of GDM.

## 4. Discussion

Gestational diabetes mellitus (GDM) is the most common metabolic disease during pregnancy [48]. The prevalence of GDM is quickly rising in the context of the global obesity epidemic [48, 49]. GDM is described as carbohydrate intolerance of variable severity with onset or first detection during pregnancy [50], encompassing abnormal glucose tolerance and diabetes mellitus, which was undiagnosed prior to or began concomitantly during pregnancy [51]. Besides, epigenetic modification plays a critical role in multiple fundamental cellular processes [52], in which epigenetic alterations are involved in the early stage of metabolic diseases including GDM [53]. As a well-recognized epigenetic marker, some clues of the essential correlation of CpG methylation with GDM were presented in some studies. It has been found that DNA methylation profiles in the placenta display that aberrant patterns of CpG methylation in GDM may be involved in the progression of GDM [54]. In addition, early pregnancy peripheral blood DNA methylation is different in repeat pregnancies with the change in GDM status [55]. Deregulation of CpG methylation in adipose tissues and blood cells is correlated with gestational diabetes and neonatal outcome [56]. A study also provides pieces of evidence that placental global DNA hypermethylation is associated with GDM [57]. In this study, we identified 62 significant CpG methylation sites with the gene annotation in the GDM samples compared with the control samples from the GSE88929 of the GEO database by using the EWAS analysis, in which these CpG sites were distributed within the body region, promoter region, exon, 3′ UTR, and 5′ UTR. These data suggest that the identified CpG sites may be potentially related to the result of GDM, providing new evidence of the correlation of CpG methylation with GDM.

The etiology of GDM is complicated, with genetic and environmental factors involved in mechanistic and epidemiological studies [48]. GDM is regularly the result of *β*-cell dysfunction in a setting of chronic insulin resistance during pregnancy, and *β*-cell impairment and tissue insulin resistance represent critical components of the pathophysiology of GDM [58]. There are multiple contributors involved in the development of GDM. ATPase-associated protein participates in the progression of GDM [59]. Ras-related protein Rap1A is decreased in the GDM samples and influences insulin resistance [60]. Autophagy is enhanced in GDM patients and displays a substantial function in GDM [61]. The ROCK1/p53/NOXA axis modulates the apoptosis disorder in response to GDM [62]. Moreover, the diagnosis of GDM is critical for the early treatment of GDM to reduce the risk of adverse events [63–66]. Several biomarkers of GDM are identified, including itaconic acid, microRNAs, inflammatory markers, and PD-1 [67–70]. The HbA1c showed high sensitivity with relatively low specificity for the diagnosis of GDM in pregnant women [71, 72]. Furthermore, emerging evidence revealed the use of DNA methylation as biomarkers that could benefit the early diagnosis of GDM, improving the management of GDM and enhancing health outcomes [66]. Our GO and KEGG analyses based on the 62 CpG sites demonstrated that several important cellular processes, such as sodium:potassium−exchanging ATPase complex and Cul4A−RING E3 ubiquitin ligase complex, and many essential signaling pathways, including Type I diabetes mellitus, Ras signaling pathway, p53 signaling pathways, and autophagy, were identified in the system. It suggests that these cellular processes and signaling may play crucial roles in the development of GDM, enriching the potential mechanism of GDM progression. Besides, we found 12 related genes, including C7orf50, RASA3, PPFIA1, CASKIN1, CUL4A, POLE, MCM5, TUBGCP3, JAKMIP1, TYK2, C17orf70, and EME2, in the PPI network. It indicates that these genes may participate in the GDM progression and may serve as promising biomarkers for the GDM diagnosis. The specific effect of these genes on GDM progression is needed to investigate further.

Machine learning has the potential to be extremely useful in clinical prediction [73, 74], which is being developed to benefit the diagnosis of clinical samples [75, 76]. As a widely applied machine learning algorithm, SVM is reported to be used in the investigation of GDM. It has been reported that SVM is applied in the identification of diagnostic biomarkers in patients with GDM based on transcriptome gene expression and methylation analysis [7]. The artificial immune recognition system and SVM model contribute to predicting GDM [77]. In the present study, our EWAS analysis identified that 6 CpG sites, including cg00922748, cg05216211, cg05376185, cg06617468, cg17097119, and cg22385669, were located in the promoter region. Among the 6 CpG sites, cg22385669 is located in the promoter region of AQR, which encodes the spliceosomal intron binding protein [78]. Song et al. revealed that AQR was related to type 2 diabetes mellitus and involved in the regulation of glucose metabolism-related pathways [79]. AQR deletion could not only promote the uptake of glucose but also restore the sensitivity of insulin. cg00922748 is located on NRIP2, a member of the aspartic protease family [80]. Although there is no evidence on the association between NRIP2 and GDM, it was shown that decreased NRIP1 expression was able to affect the glucose metabolism [81]. However, the association of the corresponding genes of the left 4 CpG sites with GDM or glucose metabolism remains unclear, which still needs further investigation. We established an SVM diagnostic model, in which the predictor variable was the *β*-values of the identified 6 CpG sites in GSE88929, and the outcome variable was the occurrences of GDM. Surprisingly, the AUC values of the training set and testing set in the model were 0.8138 and 0.7576, respectively. Moreover, the AUC value of the independent validation set of GSE102177 was 0.6667. Our data suggest that this SVM model is reliable and accurate for the diagnosis of GDM. Meanwhile, the models need to be further optimized for improving the performance in GDM prediction.

## 5. Conclusion

In conclusion, this study identified potential diagnostic CpG biomarkers in patients with gestational diabetes mellitus by the combination of epigenome-wide association study and SVM model. The SVM model based on the identified 6 CpG sites reliably predicted the occurrence of GDM in patients, benefiting the diagnosis of GDM. Our finding provides new insights into the cross-application of EWAS and machine learning to explore the correlation of DNA methylation with GDM development.

## Data Availability

Our data, DNA methylation data, and related clinical information were obtained from the GEO database (http://www.ncbi.nlm.nih.gov/geo/).

## Abbreviations

GDM: Gestational diabetes mellitus
EWAS: Epigenome-wide association study
GO: Gene Ontology
KEGG: Kyoto Encyclopedia of Genes and Genomes
PPI: Protein-protein interaction
SVM: Support vector machine
ROC: Receiver operating characteristic
AUC: Area under the curve.

## Supplementary Materials

Supplementary 1 Fig. S1: PPI network construction. (A) The protein-protein interaction (PPI) analysis was constructed in the STRING online database (https://string-db.org/cgi/input.pl) and Cytoscape software based on the 60 identified CpG sites-related genes. The PPI network of 12 CpG methylation-related genes was shown.

Supplementary 2 Table S1: the gene annotation of 62 identified CpG sites-related genes. Table S2: the information of GO and KEGG analyses based on the identified CpG site-related genes. Table S3: the sample information of the training set and testing set in the GSE88929 dataset.
