# Supplementary figures and images for: Identification of Diagnostic CpG Signatures in Patients with Gestational Diabetes Mellitus via Epigenome-Wide Association Study Integrated with Machine Learning
Source: Biomed Res Int. 2021 May 19;2021:1984690. doi: 10.1155/2021/1984690 (PMC8162250; doi:10.1155/2021/1984690)

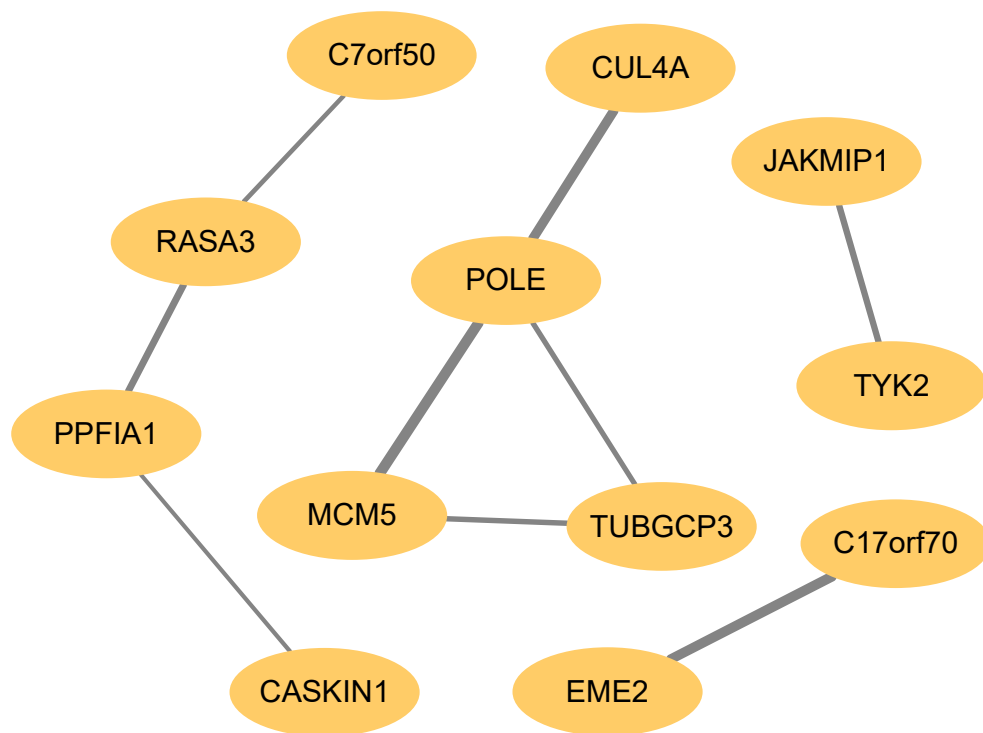

Supplement: Supplementary 1 — Fig. S1: PPI network construction. (A) The protein-protein interaction (PPI) analysis was constructed in the STRING online database (https://string-db.org/cgi/input.pl) and Cytoscape software based on the 60 identified CpG sites-related genes. The PPI network of 12 CpG methylation-related genes was shown. [file 1984690.f1.pdf]
