# Supplementary material for: Identification of Diagnostic CpG Signatures in Patients with Gestational Diabetes Mellitus via Epigenome-Wide Association Study Integrated with Machine Learning
Source: Biomed Res Int. 2021 May 19;2021:1984690. doi: 10.1155/2021/1984690 (PMC8162250; doi:10.1155/2021/1984690)
Supplement: Supplementary 2 — Table S1: the gene annotation of 62 identified CpG sites-related genes. Table S2: the information of GO and KEGG analyses based on the identified CpG site-related genes. Table S3: the sample information of the training set and testing set in the GSE88929 dataset. [file 1984690.f2.zip › Table S1.docx]

Table S1 The gene annotation of 62 identifed CpG sites-related genes

| CPG.Labels | Gene | CPG.Labels | Gene |
| --- | --- | --- | --- |
| cg00504226 | AGPAT3 | cg12170299 | FAM83C |
| cg00590639 | TYK2 | cg12388397 | C17orf70 |
| cg00922748 | NRIP2 | cg13206735 | ATP9B |
| cg01547142 | MCM5 | cg13369686 | PTPRN2 |
| cg01940439 | SFRS8 | cg13429812 | CUL4A |
| cg01975495 | SERPINE1 | cg13481374 | KIF24 |
| cg02049404 | CASKIN1 | cg13596910 | C17orf65 |
| cg02381965 | TTC15 | cg14273093 | FAM101A |
| cg03193865 | BOP1 | cg14851271 | HLA-DMA |
| cg03480698 | PTPRN2 | cg16561957 | CDC42BPB |
| cg03932459 | RGL2 | cg16673806 | KDM3B |
| cg05115233 | RASA3 | cg16942223 | JAKMIP1 |
| cg05216211 | MIR1185-1 | cg16956999 | POLE |
| cg05376185 | MOBKL2C | cg16991298 | ATP1B2 |
| cg06609583 | ATP11A | cg17097119 | DEFB133 |
| cg06617468 | NAP1L5 | cg17442961 | RPTOR |
| cg06753985 | EME2 | cg17514098 | SCARB1 |
| cg06898946 | SFXN5 | cg17745122 | IRX3 |
| cg06903151 | PPFIA1 | cg18945794 | RGS12 |
| cg07627464 | LMTK2 | cg19189310 | RPS10 |
| cg09248944 | CD302 | cg20595750 | DIP2C |
| cg09626833 | TUBGCP3 | cg21113740 | ELTD1 |
| cg09642777 | TMEM62 | cg22385669 | AQR |
| cg09668072 | SEPT9 | cg24280439 | TBCD |
| cg09676606 | SYCP3 | cg24458329 | CASP9 |
| cg10119288 | ZCCHC14 | cg24514230 | CHRNA10 |
| cg10206452 | KIAA1026 | cg25381331 | SLC1A7 |
| cg10239991 | HDAC4 | cg25592858 | INF2 |
| cg11253191 | C17orf70 | cg25815027 | RASAL1 |
| cg11541678 | MLC1 | cg26786253 | ZZEF1 |
| cg11995490 | C7orf50 | cg26850624 | AHRR |
