# Supplementary material for: Identification of Diagnostic CpG Signatures in Patients with Gestational Diabetes Mellitus via Epigenome-Wide Association Study Integrated with Machine Learning
Source: Biomed Res Int. 2021 May 19;2021:1984690. doi: 10.1155/2021/1984690 (PMC8162250; doi:10.1155/2021/1984690)
Supplement: Supplementary 2 — Table S1: the gene annotation of 62 identified CpG sites-related genes. Table S2: the information of GO and KEGG analyses based on the identified CpG site-related genes. Table S3: the sample information of the training set and testing set in the GSE88929 dataset. [file 1984690.f2.zip › Table S2.docx]

Table S2 The information of GO analysis based on the identified CpG sites-related genes

| ONTOLOGY | ID | Description | GeneRatio | BgRatio | pvalue | p.adjust | qvalue | geneID | Count |
| --- | --- | --- | --- | --- | --- | --- | --- | --- | --- |
| CC | GO:0042641 | actomyosin | 3/47 | 66/18698 | 0.000609557 | 0.052147542 | 0.045185611 | 10801/9759/9578 | 3 |
| CC | GO:0031235 | intrinsic component of the cytoplasmic  side of the plasma membrane | 2/47 | 15/18698 | 0.000635946 | 0.052147542 | 0.045185611 | 22821/8437 | 2 |
| CC | GO:0005874 | microtubule | 5/47 | 408/18698 | 0.003487016 | 0.190623535 | 0.165174436 | 10426/10801/ 347240/ 152789/6904 | 5 |
| CC | GO:0055037 | recycling endosome | 3/47 | 165/18698 | 0.008233897 | 0.259017433 | 0.224437442 | 23250/22853/ 23209 | 3 |
| CC | GO:0009898 | cytoplasmic side of plasma membrane | 3/47 | 176/18698 | 0.009814403 | 0.259017433 | 0.224437442 | 7297/22821/8437 | 3 |
| CC | GO:0005765 | lysosomal membrane | 4/47 | 355/18698 | 0.011992174 | 0.259017433 | 0.224437442 | 23250/3108/ 57521/949 | 4 |
| CC | GO:0098852 | lytic vacuole membrane | 4/47 | 355/18698 | 0.011992174 | 0.259017433 | 0.224437442 | 23250/3108/ 57521/949 | 4 |
| CC | GO:0098562 | cytoplasmic side of membrane | 3/47 | 199/18698 | 0.013657322 | 0.259017433 | 0.224437442 | 7297/22821/8437 | 3 |
| CC | GO:0005901 | caveola | 2/47 | 78/18698 | 0.016449907 | 0.259017433 | 0.224437442 | 23209/949 | 2 |
| CC | GO:0032153 | cell division site | 2/47 | 81/18698 | 0.01766393 | 0.259017433 | 0.224437442 | 10426/10801 | 2 |
| CC | GO:0005902 | microvillus | 2/47 | 83/18698 | 0.018493865 | 0.259017433 | 0.224437442 | 9936/949 | 2 |
| CC | GO:0005774 | vacuolar membrane | 4/47 | 414/18698 | 0.019979379 | 0.259017433 | 0.224437442 | 23250/3108/ 57521/949 | 4 |
| CC | GO:0000794 | condensed nuclear chromosome | 2/47 | 96/18698 | 0.024276955 | 0.259017433 | 0.224437442 | 50511/6002 | 2 |
| CC | GO:0044853 | plasma membrane raft | 2/47 | 98/18698 | 0.025224591 | 0.259017433 | 0.224437442 | 23209/949 | 2 |
| CC | GO:0005890 | sodium:potassium-exchanging  ATPase complex | 1/47 | 11/18698 | 0.027312326 | 0.259017433 | 0.224437442 | 482 | 1 |
| CC | GO:0042555 | MCM complex | 1/47 | 11/18698 | 0.027312326 | 0.259017433 | 0.224437442 | 4174 | 1 |
| CC | GO:0031464 | Cul4A-RING E3 ubiquitin ligase complex | 1/47 | 13/18698 | 0.032199151 | 0.259017433 | 0.224437442 | 8451 | 1 |
| CC | GO:0005940 | septin ring | 1/47 | 14/18698 | 0.034633544 | 0.259017433 | 0.224437442 | 10801 | 1 |
| CC | GO:0031105 | septin complex | 1/47 | 14/18698 | 0.034633544 | 0.259017433 | 0.224437442 | 10801 | 1 |
| CC | GO:0032160 | septin filament array | 1/47 | 14/18698 | 0.034633544 | 0.259017433 | 0.224437442 | 10801 | 1 |
| CC | GO:0032156 | septin cytoskeleton | 1/47 | 15/18698 | 0.037061944 | 0.259017433 | 0.224437442 | 10801 | 1 |
| CC | GO:0090533 | cation-transporting ATPase complex | 1/47 | 15/18698 | 0.037061944 | 0.259017433 | 0.224437442 | 482 | 1 |
| CC | GO:0000800 | lateral element | 1/47 | 16/18698 | 0.039484365 | 0.259017433 | 0.224437442 | 50511 | 1 |
| CC | GO:0038201 | TOR complex | 1/47 | 16/18698 | 0.039484365 | 0.259017433 | 0.224437442 | 57521 | 1 |
| CC | GO:0042613 | MHC class II protein complex | 1/47 | 16/18698 | 0.039484365 | 0.259017433 | 0.224437442 | 3108 | 1 |
| CC | GO:0005814 | centriole | 2/47 | 129/18698 | 0.041725816 | 0.259537772 | 0.224888313 | 10426/347240 | 2 |
| CC | GO:0005892 | acetylcholine-gated channel complex | 1/47 | 18/18698 | 0.044311327 | 0.259537772 | 0.224888313 | 57053 | 1 |
| CC | GO:0042575 | DNA polymerase complex | 1/47 | 18/18698 | 0.044311327 | 0.259537772 | 0.224888313 | 5426 | 1 |
| CC | GO:0000930 | gamma-tubulin complex | 1/47 | 20/18698 | 0.049114545 | 0.277751221 | 0.240670185 | 10426 | 1 |
| MF | GO:0004012 | phospholipid-translocating  ATPase activity | 2/49 | 20/17548 | 0.001405431 | 0.228353358 | 0.191482745 | 23250/374868 | 2 |
| MF | GO:0060589 | nucleoside-triphosphatase  regulator activity | 5/49 | 359/17548 | 0.003176617 | 0.228353358 | 0.191482745 | 22821/482/6002/ 6904/8437 | 5 |
| MF | GO:0043492 | ATPase activity, coupled to  movement of  substances | 3/49 | 129/17548 | 0.005588202 | 0.228353358 | 0.191482745 | 23250/374868/ 482 | 3 |
| MF | GO:0005096 | GTPase activator activity | 4/49 | 286/17548 | 0.008233899 | 0.228353358 | 0.191482745 | 22821/6002/ 6904/8437 | 4 |
| MF | GO:0005548 | phospholipid transporter activity | 2/49 | 56/17548 | 0.010685961 | 0.228353358 | 0.191482745 | 23250/374868 | 2 |
| MF | GO:0030695 | GTPase regulator activity | 4/49 | 316/17548 | 0.01157096 | 0.228353358 | 0.191482745 | 22821/6002/ 6904/8437 | 4 |
| MF | GO:0017048 | Rho GTPase binding | 3/49 | 173/17548 | 0.012449102 | 0.228353358 | 0.191482745 | 5863/9578/64423 | 3 |
| MF | GO:0004536 | deoxyribonuclease activity | 2/49 | 68/17548 | 0.015475117 | 0.228353358 | 0.191482745 | 197342/5426 | 2 |
| MF | GO:0015631 | tubulin binding | 4/49 | 351/17548 | 0.016441609 | 0.228353358 | 0.191482745 | 10426/347240/ 152789/6904 | 4 |
| MF | GO:0000287 | magnesium ion binding | 3/49 | 196/17548 | 0.017351282 | 0.228353358 | 0.191482745 | 23250/374868/ 9578 | 3 |
| MF | GO:0140097 | catalytic activity, acting on DNA | 3/49 | 197/17548 | 0.017585582 | 0.228353358 | 0.191482745 | 4174/197342/5426 | 3 |
| MF | GO:0000182 | rDNA binding | 1/49 | 10/17548 | 0.027582123 | 0.228353358 | 0.191482745 | 57521 | 1 |
| MF | GO:0008296 | 3'-5'-exodeoxyribonuclease  activity | 1/49 | 10/17548 | 0.027582123 | 0.228353358 | 0.191482745 | 5426 | 1 |
| MF | GO:0017153 | sodium:dicarboxylate symporter  activity | 1/49 | 10/17548 | 0.027582123 | 0.228353358 | 0.191482745 | 6512 | 1 |
| MF | GO:0031702 | type 1 angiotensin receptor binding | 1/49 | 10/17548 | 0.027582123 | 0.228353358 | 0.191482745 | 7297 | 1 |
| MF | GO:0051011 | microtubule minus-end binding | 1/49 | 10/17548 | 0.027582123 | 0.228353358 | 0.191482745 | 10426 | 1 |
| MF | GO:0031078 | histone deacetylase activity  (H3-K14 specific) | 1/49 | 11/17548 | 0.030298993 | 0.228353358 | 0.191482745 | 9759 | 1 |
| MF | GO:0031701 | angiotensin receptor binding | 1/49 | 11/17548 | 0.030298993 | 0.228353358 | 0.191482745 | 7297 | 1 |
| MF | GO:0032041 | NAD-dependent histone deacetylase  activity (H3-K14 specific) | 1/49 | 11/17548 | 0.030298993 | 0.228353358 | 0.191482745 | 9759 | 1 |
| MF | GO:0015238 | drug transmembrane transporter  activity | 2/49 | 99/17548 | 0.031204002 | 0.228353358 | 0.191482745 | 94097/6512 | 2 |
| MF | GO:0005391 | sodium:potassium-exchanging  ATPase activity | 1/49 | 12/17548 | 0.033008428 | 0.228353358 | 0.191482745 | 482 | 1 |
| MF | GO:0008556 | potassium-transporting ATPase activity | 1/49 | 12/17548 | 0.033008428 | 0.228353358 | 0.191482745 | 482 | 1 |
| MF | GO:0032454 | histone demethylase activity  (H3-K9 specific) | 1/49 | 13/17548 | 0.035710445 | 0.228353358 | 0.191482745 | 51780 | 1 |
| MF | GO:0099094 | ligand-gated cation channel activity | 2/49 | 109/17548 | 0.037203539 | 0.228353358 | 0.191482745 | 22821/57053 | 2 |
| MF | GO:0016887 | ATPase activity | 4/49 | 457/17548 | 0.038320973 | 0.228353358 | 0.191482745 | 23250/374868/ 347240/482 | 4 |
| MF | GO:0046873 | metal ion transmembrane  transporter activity | 4/49 | 457/17548 | 0.038320973 | 0.228353358 | 0.191482745 | 22821/482/ 57053/6512 | 4 |
| MF | GO:0003688 | DNA replication origin binding | 1/49 | 14/17548 | 0.038405067 | 0.228353358 | 0.191482745 | 4174 | 1 |
| MF | GO:0005313 | L-glutamate transmembrane transporter  activity | 1/49 | 14/17548 | 0.038405067 | 0.228353358 | 0.191482745 | 6512 | 1 |
| MF | GO:0015172 | acidic amino acid transmembrane  transporter activity | 1/49 | 14/17548 | 0.038405067 | 0.228353358 | 0.191482745 | 6512 | 1 |
| MF | GO:0097200 | cysteine-type endopeptidase activity  involved in  execution phase of apoptosis | 1/49 | 14/17548 | 0.038405067 | 0.228353358 | 0.191482745 | 842 | 1 |
| MF | GO:0008017 | microtubule binding | 3/49 | 268/17548 | 0.038776132 | 0.228353358 | 0.191482745 | 10426/347240/ 152789 | 3 |
| MF | GO:0005545 | 1-phosphatidylinositol binding | 1/49 | 15/17548 | 0.041092312 | 0.228353358 | 0.191482745 | 949 | 1 |
| MF | GO:0030955 | potassium ion binding | 1/49 | 15/17548 | 0.041092312 | 0.228353358 | 0.191482745 | 9759 | 1 |
| MF | GO:0050811 | GABA receptor binding | 1/49 | 15/17548 | 0.041092312 | 0.228353358 | 0.191482745 | 152789 | 1 |
| MF | GO:0005001 | transmembrane receptor protein tyrosine  phosphatase activity | 1/49 | 16/17548 | 0.0437722 | 0.228353358 | 0.191482745 | 5799 | 1 |
| MF | GO:0017136 | NAD-dependent histone  deacetylase activity | 1/49 | 16/17548 | 0.0437722 | 0.228353358 | 0.191482745 | 9759 | 1 |
| MF | GO:0019198 | transmembrane receptor  protein phosphatase  activity | 1/49 | 16/17548 | 0.0437722 | 0.228353358 | 0.191482745 | 5799 | 1 |
| MF | GO:0022848 | acetylcholine-gated cation-selective  channel  activity | 1/49 | 16/17548 | 0.0437722 | 0.228353358 | 0.191482745 | 57053 | 1 |
| MF | GO:0023026 | MHC class II protein complex binding | 1/49 | 16/17548 | 0.0437722 | 0.228353358 | 0.191482745 | 3108 | 1 |
| MF | GO:0034185 | apolipoprotein binding | 1/49 | 16/17548 | 0.0437722 | 0.228353358 | 0.191482745 | 949 | 1 |
| MF | GO:0097153 | cysteine-type endopeptidase activity  involved  in apoptotic process | 1/49 | 16/17548 | 0.0437722 | 0.228353358 | 0.191482745 | 842 | 1 |
| MF | GO:0005262 | calcium channel activity | 2/49 | 120/17548 | 0.044269341 | 0.228353358 | 0.191482745 | 22821/57053 | 2 |
| MF | GO:0005283 | amino acid:sodium symporter activity | 1/49 | 17/17548 | 0.046444751 | 0.228353358 | 0.191482745 | 6512 | 1 |
| MF | GO:0008329 | signaling pattern recognition receptor  activity | 1/49 | 17/17548 | 0.046444751 | 0.228353358 | 0.191482745 | 949 | 1 |
| MF | GO:0030169 | low-density lipoprotein particle binding | 1/49 | 17/17548 | 0.046444751 | 0.228353358 | 0.191482745 | 949 | 1 |
| MF | GO:0030228 | lipoprotein particle receptor activity | 1/49 | 17/17548 | 0.046444751 | 0.228353358 | 0.191482745 | 949 | 1 |
| MF | GO:0034979 | NAD-dependent protein deacetylase  activity | 1/49 | 17/17548 | 0.046444751 | 0.228353358 | 0.191482745 | 9759 | 1 |
| MF | GO:0038187 | pattern recognition receptor activity | 1/49 | 17/17548 | 0.046444751 | 0.228353358 | 0.191482745 | 949 | 1 |
