# Supplementary material for: Identification of Diagnostic CpG Signatures in Patients with Gestational Diabetes Mellitus via Epigenome-Wide Association Study Integrated with Machine Learning
Source: Biomed Res Int. 2021 May 19;2021:1984690. doi: 10.1155/2021/1984690 (PMC8162250; doi:10.1155/2021/1984690)
Supplement: Supplementary 2 — Table S1: the gene annotation of 62 identified CpG sites-related genes. Table S2: the information of GO and KEGG analyses based on the identified CpG site-related genes. Table S3: the sample information of the training set and testing set in the GSE88929 dataset. [file 1984690.f2.zip › Table S3-revised.docx]

Table S3 The sample information of the training set and testing set in GSE88929 dataset

| Sample | Type |
| --- | --- |
| GSM2355356 | Training Set |
| GSM2355382 | Training Set |
| GSM2355322 | Training Set |
| GSM2355388 | Training Set |
| GSM2355331 | Training Set |
| GSM2355429 | Training Set |
| GSM2355317 | Training Set |
| GSM2355413 | Training Set |
| GSM2355327 | Training Set |
| GSM2355379 | Training Set |
| GSM2355362 | Training Set |
| GSM2355324 | Training Set |
| GSM2355354 | Training Set |
| GSM2355395 | Training Set |
| GSM2355425 | Training Set |
| GSM2355371 | Training Set |
| GSM2355332 | Training Set |
| GSM2355378 | Training Set |
| GSM2355338 | Training Set |
| GSM2355417 | Training Set |
| GSM2355359 | Training Set |
| GSM2355350 | Training Set |
| GSM2355333 | Training Set |
| GSM2355440 | Training Set |
| GSM2355433 | Training Set |
| GSM2355412 | Training Set |
| GSM2355330 | Training Set |
| GSM2355329 | Training Set |
| GSM2355326 | Training Set |
| GSM2355368 | Training Set |
| GSM2355346 | Training Set |
| GSM2355318 | Training Set |
| GSM2355428 | Training Set |
| GSM2355389 | Training Set |
| GSM2355319 | Training Set |
| GSM2355370 | Training Set |
| GSM2355342 | Training Set |
| GSM2355335 | Training Set |
| GSM2355328 | Training Set |
| GSM2355415 | Training Set |
| GSM2355421 | Training Set |
| GSM2355439 | Training Set |
| GSM2355355 | Training Set |
| GSM2355424 | Training Set |
| GSM2355430 | Training Set |
| GSM2355436 | Training Set |
| GSM2355397 | Training Set |
| GSM2355419 | Training Set |
| GSM2355334 | Training Set |
| GSM2355391 | Training Set |
| GSM2355393 | Training Set |
| GSM2355442 | Training Set |
| GSM2355325 | Training Set |
| GSM2355403 | Training Set |
| GSM2355374 | Training Set |
| GSM2355386 | Training Set |
| GSM2355398 | Training Set |
| GSM2355392 | Training Set |
| GSM2355351 | Training Set |
| GSM2355336 | Training Set |
| GSM2355399 | Training Set |
| GSM2355414 | Training Set |
| GSM2355426 | Training Set |
| GSM2355337 | Training Set |
| GSM2355381 | Training Set |
| GSM2355358 | Training Set |
| GSM2355316 | Testing Set |
| GSM2355320 | Testing Set |
| GSM2355321 | Testing Set |
| GSM2355323 | Testing Set |
| GSM2355339 | Testing Set |
| GSM2355340 | Testing Set |
| GSM2355341 | Testing Set |
| GSM2355343 | Testing Set |
| GSM2355344 | Testing Set |
| GSM2355345 | Testing Set |
| GSM2355347 | Testing Set |
| GSM2355348 | Testing Set |
| GSM2355349 | Testing Set |
| GSM2355352 | Testing Set |
| GSM2355353 | Testing Set |
| GSM2355357 | Testing Set |
| GSM2355360 | Testing Set |
| GSM2355361 | Testing Set |
| GSM2355363 | Testing Set |
| GSM2355364 | Testing Set |
| GSM2355365 | Testing Set |
| GSM2355366 | Testing Set |
| GSM2355367 | Testing Set |
| GSM2355369 | Testing Set |
| GSM2355372 | Testing Set |
| GSM2355373 | Testing Set |
| GSM2355375 | Testing Set |
| GSM2355376 | Testing Set |
| GSM2355377 | Testing Set |
| GSM2355380 | Testing Set |
| GSM2355383 | Testing Set |
| GSM2355384 | Testing Set |
| GSM2355385 | Testing Set |
| GSM2355387 | Testing Set |
| GSM2355390 | Testing Set |
| GSM2355394 | Testing Set |
| GSM2355396 | Testing Set |
| GSM2355400 | Testing Set |
| GSM2355401 | Testing Set |
| GSM2355402 | Testing Set |
| GSM2355404 | Testing Set |
| GSM2355405 | Testing Set |
| GSM2355406 | Testing Set |
| GSM2355407 | Testing Set |
| GSM2355408 | Testing Set |
| GSM2355409 | Testing Set |
| GSM2355410 | Testing Set |
| GSM2355411 | Testing Set |
| GSM2355416 | Testing Set |
| GSM2355418 | Testing Set |
| GSM2355420 | Testing Set |
| GSM2355422 | Testing Set |
| GSM2355423 | Testing Set |
| GSM2355427 | Testing Set |
| GSM2355431 | Testing Set |
| GSM2355432 | Testing Set |
| GSM2355434 | Testing Set |
| GSM2355435 | Testing Set |
| GSM2355437 | Testing Set |
| GSM2355438 | Testing Set |
| GSM2355441 | Testing Set |
| GSM2355443 | Testing Set |
| GSM2355444 | Testing Set |
| GSM2355445 | Testing Set |
| GSM2355446 | Testing Set |
| GSM2355447 | Testing Set |
